# Control of hydrogen concentrations by microbial sulfate reduction in two contrasting anoxic coastal sediments

**DOI:** 10.3389/fmicb.2024.1455857

**Published:** 2024-11-12

**Authors:** Gage R. Coon, Leketha C. Williams, Adrianna Matthews, Roberto Diaz, Richard T. Kevorkian, Douglas E. LaRowe, Andrew D. Steen, Laura L. Lapham, Karen G. Lloyd

**Affiliations:** ^1^Department of Microbiology, The University of Tennessee, Knoxville, Knoxville, TN, United States; ^2^Department of Earth Sciences, University of Southern California, Los Angeles, CA, United States; ^3^Department of Earth and Planetary Sciences, The University of Tennessee, Knoxville, Knoxville, TN, United States; ^4^Department Marine and Environmental Biology, University of Southern California, Los Angeles, CA, United States; ^5^Chesapeake Biological Laboratory, University of Maryland Center for Environmental Science, Solomons, MD, United States

**Keywords:** hydrogen, methane, AOM, methanogenesis, sulfate reduction, thermodynamics, organic matter, marine sediment

## Abstract

**Introduction:**

Molecular hydrogen is produced by the fermentation of organic matter and consumed by organisms including hydrogenotrophic methanogens and sulfate reducers in anoxic marine sediment. The thermodynamic feasibility of these metabolisms depends strongly on organic matter reactivity and hydrogen concentrations; low organic matter reactivity and high hydrogen concentrations can inhibit fermentation so when organic matter is poor, fermenters might form syntrophies with methanogens and/or sulfate reducers who alleviate thermodynamic stress by keeping hydrogen concentrations low and tightly controlled. However, it is unclear how these metabolisms effect porewater hydrogen concentrations in natural marine sediments of different organic matter reactivities.

**Methods:**

We measured aqueous concentrations of hydrogen, sulfate, methane, dissolved inorganic carbon, and sulfide with high-depth-resolution and 16S rRNA gene assays in sediment cores with low carbon reactivity in White Oak River (WOR) estuary, North Carolina, and those with high carbon reactivity in Cape Lookout Bight (CLB), North Carolina. We calculated the Gibbs energies of sulfate reduction and hydrogenotrophic methanogenesis.

**Results:**

Hydrogen concentrations were significantly higher in the sulfate reduction zone at CLB than WOR (mean: 0.716 vs. 0.437 nM H_2_) with highly contrasting hydrogen profiles. At WOR, hydrogen was extremely low and invariant (range: 0.41–0.52 nM H_2_) in the upper 15 cm. Deeper than 15 cm, hydrogen became more variable (range: 0.312–2.56 nM H_2_) and increased until methane production began at ~30 cm. At CLB, hydrogen was highly variable in the upper 15 cm (range: 0.08–2.18 nM H_2_). Ratios of inorganic carbon production to sulfate consumption show AOM drives sulfate reduction in WOR while degradation of organics drive sulfate reduction in CLB.

**Discussion:**

We conclude more reactive organic matter increases hydrogen concentrations and their variability in anoxic marine sediments. In our AOM-dominated site, WOR, sulfate reducers have tight control on hydrogen via consortia with fermenters which leads to the lower observed variance due to interspecies hydrogen transfer. After sulfate depletion, hydrogen accumulates and becomes variable, supporting methanogenesis. This suggests that CLB’s more reactive organic matter allows fermentation to occur without tight metabolic coupling of fermenters to sulfate reducers, resulting in high and variable porewater hydrogen concentrations that prevent AOM from occurring through reverse hydrogenotrophic methanogenesis.

## 1 Introduction

Methane is a potent greenhouse gas that has more than doubled in the atmosphere since the pre-industrial era (Etheridge et al., 1998; US Department of Commerce, N, 2023). Therefore, it is important to understand what controls its sources and sinks, both natural and anthropogenic. Of the ~85 Tg/yr of methane produced in marine sediments, only about one-tenth of this methane is released into the overlying water column, because most of it is removed with sulfate-dependent anaerobic oxidation of methane (AOM) (Reeburgh, 2007), a microbially mediated process whereby upward-diffusing methane is oxidized to carbon dioxide while sulfate is reduced to sulfide in anoxic marine sediments. AOM communities can also use other electron acceptors like nitrate, nitrite, and metal ions to consume methane (Beal et al., 2009; Haroon et al., 2013; Muyzer and Stams, 2008; Timmers et al., 2017; Zhang et al., 2022), although these alternatives have not been shown to be quantitatively important in anoxic sulfate-rich marine sediments.

Hydrogen is a key electron donor that controls the fluxes of important compounds like methane and sulfate in anoxic marine sediments. Hydrogen is produced, along with volatile fatty acids, through the fermentation of a wide range of organic carbon molecules (LaRowe et al., 2020b). The most prevalent methanogenesis pathway in marine sediments—hydrogenotrophic methanogenesis—uses this hydrogen to reduce carbon dioxide to methane (Liu and Whitman, 2008). AOM in marine sediments has been shown to occur through a reversal of hydrogenotrophic methanogenesis with the direction controlled by hydrogen concentrations due to the power of four effect on the energetics in the reversible Equation 1 (Coon et al., 2023; Hoehler et al., 1994, 1998; Timmers et al., 2017):

(1) $${CO}_{2}+4H_{2}↔{CH}_{4}+2H_{2}O$$

The formation of syntrophic partnerships between fermenters and sulfate reducers that use metabolic byproducts to prevent the buildup of inhibitory end products can keep hydrogen concentrations low enough to promote the favorability of both fermentation and AOM (Morris et al., 2013). For example, uncultured anaerobic methanotrophic archaea (*ANME*) form consortia with sulfate reducing bacteria (SRB) where the sulfate reducers gain the electrons which promotes the oxidation of methane by *ANME* through net Equation 2:

(2) $${CH}_{4}+{{\mathrm{SO}}_{4}}^{2-}\to {{HCO}_{3}}^{-}+{HS}^{-}+H_{2}O$$

This reaction is the reverse of reaction 1 (or reaction 6, which more accurately reflects that the system uses bicarbonate) but written as the net reaction including sulfate reduction to reflect the consortia dynamics (Hoehler and Alperin, 1996). AOM through reverse hydrogenotrophic methanogenesis creates hydrogen as an intermediate that can be used by SRB as an electron donor (Hoehler et al., 1994; Knittel and Boetius, 2009). Therefore, if SRB form tight consortia with hydrogen-producing fermenters, they can theoretically keep hydrogen concentrations consistently low, preventing methanogenesis and enabling AOM. However, if hydrogen production via fermentation exceeds its consumption via sulfate reduction, hydrogen can accumulate, preventing AOM through reverse hydrogenotrophic methanogenesis. In environments with a high content of labile organic matter, such as anaerobic sludge reactors, hydrogen concentrations can even be high enough to support simultaneous methane production and sulfate reduction (Lovley et al., 1982; Santegoeds et al., 1999; Timmers et al., 2015). Another way to reduce sulfate is through organoclastic sulfate reduction (OSR), the fermentation of organics, through the simplified net Equation 3:

(3) $${2CH}_{2}O+{{\mathrm{SO}}_{4}}^{2-}\to {{\mathrm{2HCO}}_{3}}^{-}+{HS}^{-}+H^{+}$$

We can test if sulfate reduction is carried out through syntrophic relationships with AOM or through OSR since the amount of dissolved inorganic carbon (DIC) produced is different for each process (Yang et al., 2008). Thus, ∆DIC:∆SO_4_^2−^ increases with a 1:1 ratio for AOM and a 2:1 ratio for OSR assuming the nominal oxidation state of organic carbon (NOSC) is 0 (see LaRowe and Van Cappellen, 2011). However, if the organic matter has a non-neutral NOSC, then the ratios will be slightly different. The use of just hydrogen to reduce sulfate does not yield DIC, and therefore is not considered. AOM can also be demonstrated by an upward-curved profile of methane concentrations with depth; a linear increase in methane with depth instead signifies no net AOM or methanogenesis (Martens and Goldhaber, 1978).

The Gibbs energy function can be used to calculate the thermodynamic favorability of metabolic reactions based on the surrounding environment—(see Amend and LaRowe, 2019). It has been proposed that the generated energy of a reaction must be at least one-third to one-fifth of the energy needed to convert ADP into ATP depending on the number of ion binding sites on the ATP synthases *c* ring (Mayer and Müller, 2014; Müller and Hess, 2017). The minimum Gibbs energy change, ∆*G_min_*, for this minimum biological energy quantum (BEQ) is often quoted as −20 kJ/mol (Schink, 1997), though this value has been proposed to be lower for substrate level phosphorylation (Müller and Hess, 2017). In anoxic sediment, apparent ∆*G_min_* values have been calculated as −19.1 kJ/mol SO_4_^2−^ for sulfate reducers and −10.6 kJ/mol CO_2_ for hydrogenotrophic methanogenic archaea (Hoehler et al., 2001). ∆*G_r_* values greater than the ∆*G_min_* (more positive than −10 to −20 kJ/mol) would inhibit microbial catalysis of catabolic reactions in either direction. In the case of Reaction 1, this means ∆*G_r_* values > −10 kJ/mol theoretically prevent biological production of methane (Hoehler et al., 1994).

Despite the critical importance of hydrogen concentrations, few studies measure it because it cannot be preserved from natural samples as easily as other dissolved gases (Hoehler et al., 1998, 2001; Lin et al., 2012; Lovley, 1985; Lovley et al., 1982). This is because hydrogen reacts so quickly that by the time a sediment sample is placed into a gas impermeable vial and capped, the hydrogen concentrations have already changed. The solution is to place sediment, avoiding disturbance and maintaining sediment structure as much as possible, into a glass serum vial, capping with a thick butyl stopper, purging the head space with an anoxic gas like N_2_, letting the hydrogen concentrations re-equilibrate over a few days, and then measuring the hydrogen partial pressure in the headspace (Hoehler et al., 1994).

To understand whether organic-rich marine sediments have higher hydrogen concentrations, preventing AOM through ∆*G_r_* limitations, we collected cores from two sites: Cape Lookout Bight, NC, (CLB, organic-rich) and White Oak River estuary, NC (WOR, organic-poor). CLB has been shown to have more labile organic matter than WOR via a higher sedimentation rate—0.25 cm/yr. for WOR (Benninger and Martens, 1983) vs. 10.3 cm/yr in CLB (Martens and Klump, 1984) and via reactive organic carbon input values—67 mol/m^2^*yr for CLB vs. 2.7 mol/m^2^*yr for WOR (Martens et al., 1998). Previous work has shown that methane removal through AOM does not occur in the upper sulfate-rich sediments of organic-rich CLB, whereas it does occur in the upper sulfate-rich sediments of organic-poor WOR (Hoehler et al., 1994; Lloyd et al., 2011; Martens et al., 1998). We therefore hypothesize that hydrogen concentrations would be higher in CLB than in WOR. We further hypothesize that these differences do not correspond to the presence or absence of uncultured clades of microbes called *ANME* that have been shown to mediate AOM, since they are commonly found in sediments and enrichments that produce methane as well as those that consume methane (Kevorkian et al., 2021, 2022; Lloyd et al., 2011; Yoshinaga et al., 2014).

## 2 Methods

### 2.1 Field sampling

Three duplicate cores were collected from Cape Lookout Bight (CLB), North Carolina, USA, 34° 37′ 01.1” N, 76° 32′ 54.4” W on June 7th, 2023. A fourth core was collected in October of 2013 from the same site. Sediment was 6.25 m below the water surface at an assumed pressure of ~1 atm. The CLB collection site salinity was assumed to be 35‰ and surface water temperature was measured as 24°C. Previously measured pH of the sediment was 7.2 (Hoehler et al., 1994, 1998). A sediment core from White Oak River (WOR) estuary, North Carolina, USA, was collected from 34° 44′ 29.4” N, 77° 7′ 26.4” W in May 2019. The collection site salinity varies tidally; 18.9‰ and a temperature of 28.5°C was used for calculations from Kelley et al. (1990). Cores were sectioned in 2 cm intervals for CLB and 1 cm intervals for WOR, with subsamples from each layer removed in the ways described below for different measurements. Of these measurements, only methane concentration, hydrogen, and porosity were measured for the WOR, since the other measurements have been published from similar cores from this site over many years (Kelley et al., 1990; Martens et al., 1998; Lloyd et al., 2011; Lloyd et al., 2020; Kevorkian et al., 2021). These studies show consistent shapes of the geochemical downcore curves over time, such that knowing the depth of methane increase in a core allows the estimation of the other parameters based on previous measurements.

For microscopy, 1 mL of fresh sediment was taken in a syringe and added to 2 mL O-ring cap tubes with 500 μL of 3–4% paraformaldehyde (PFA) diluted in phosphate-buffered saline (PBS). The sediments were weighed and stored at 4°C. For porosity, 3 mL of sediment was placed in pre-weighed glass serum vials and the wet mass was recorded. Porewater was collected by centrifuging 15 mL of sediment for 5 min and filtering through a 0.2 μm syringe filter. For sulfate, 0.7 μL of porewater was stored in 100 μL of 10% HCl. For sulfide, 1 mL of porewater was stored in 250 μL of 1% ZnCl_2_. The remaining porewater was stored in pre-weighed and evacuated glass serum vials for measuring DIC. The mass of DIC porewater was recorded. For methane concentration and δ^13^C-CH_4_, 3–4 mL of sediment was added to a glass serum vial containing 1 mL of 0.1 M KOH (enough to make pH ~ 8), capped with rubber stoppers, shaken, and stored upside down at room temperature. For hydrogen, 3 mL of sediment was collected while trying to preserve the layering and orientation of the sediment and added to an empty glass serum vial, capped with squishy butyl rubber stoppers (Rubber BV, Hilversum, NL, USA) to minimize hydrogen loss, and evacuated until flushed with O_2_-scrubbed N_2_ gas once back to the University of Tennessee, Knoxville, 2 days later.

### 2.2 Porosity

Sediment water content was calculated by drying the uncapped vials at 55°C for 2 weeks. The water loss was normalized as a fraction of the wet sediment mass. Porosity (*Φ*) was calculated from Equation 4:

(4) $$\Phi =\frac{w*\rho_{sm}}{\rho_{sm}*w+\left(1-w\right)*\rho_{pw}}$$

where *w* is sediment water content, *ρ_sm_* is solid matter density, and *ρ_pw_* is the porewater density; *ρ_sm_* and *ρ_pw_* were assumed to be 2.5 and 1.025 g/cm^3^, respectively.

For depths 0–33 cm, outliers were identified if greater than the third quartile plus 1.5 times the interquartile range or if less than the first quartile minus 1.5 times the interquartile range (*n* = 5). The average of the three adjacent values replaced the outlying porosity value. For depths 33–51 cm, outliers were identified if they were greater than three standard deviations from the average of the surrounding depths. If so, the average of the surrounding depths replaced the outlying porosity value. Supplementary Table S1 lists the porosity outliers that were recalculated to give porosity values used in further calculations.

### 2.3 Methane

Methane was measured with a gas chromatograph (GC) equipped with a flame ionized detector (GC – Agilent 7890 Network). Replicate standards ranged ±8.9% at values around the average measured ppm. Vials were shaken for at least 1 min prior to headspace sampling. 0.5 mL of headspace was injected with triplicate runs per sample. Aqueous methane concentrations [CH_4 *aq*_] were calculated in mM using Equation 5:

(5) $$\left[{CH}_{4_{aq}}\right]=\frac{{CH}_{4\;g}*V_{h}}{R*T*\Phi *V_{s}*1000}$$

where CH_4 *g*_ is the methane gas concentration in ppm converted from peak area with the standard curve, *V_h_* is the headspace volume, *R* is the universal gas constant in L*atm/mol*K, *T* is the temperature in K, *Φ* is the porosity, *V_s_* is the sediment volume, and 1,000 is the conversion factor for mM.

### 2.4 δ^13^CH_4_

δ^13^CH_4_ was measured from the same vials as methane using a cavity ringdown spectrometer (Picarro G2201i). Vials were injected with 5 mL of zero air and shaken for 2 min prior to injection. Headspace CH_4_ was diluted (5 mL headspace: 135 mL zero air) and injected directly into the spectrometer. Instrument precision was ±1‰.

### 2.5 Hydrogen

Saturation concentrations for *in situ* hydrogen was calculated as 671.8 μM for CLB and 704.9 μM for WOR to convert ppm into aqueous concentrations (Crozier and Yamamoto, 1974). Serum vials were capped and flushed with O_2_-scrubbed N_2_ gas and left to incubate at room temperature for at least 4 days. The use of squishy (easy to depress between the fingers) stoppers was tested to ensure hydrogen remains trapped in the headspace. We found that H_2_ was lost after 9 days of incubation, so we stopped all incubations at this 9 day mark (Supplementary Figure S1). After incubation, a glass syringe and metal needle were used to collect equilibrated air from the headspace without shaking the vial. Hydrogen was measured with a GC [Peak Performer 1 reducing compound photometer (RCP)]. This instrument has a precision of ±10% of the reading. Triplicates were measured except deeper than 47 cm in WOR cores due to shortage of vials in the field.

### 2.6 Microscopy

Dilutions [with phosphate-buffered saline (PBS)] of the refrigerated sediment ranged from 1:20 for 0–2 cm, 1:10 for 2–30 cm, and 1:5 for 30+ cm. 20 μL of the diluted samples were added to 5 mL of PBS with 500 μL of 5× SYBR Gold and left to incubate at room temperature in the dark for 10 min. Samples were filtered onto a 0.2 μm filter until dry then mounted with VECTASHIELD. Slides were stored at −20°C for up to 2 weeks. The slides were excited with the 38 HE GFP filter set and counted at 30 random locations on the slide with a ZEISS Axio Imager M2. The average cells counted was extrapolated for the cell concentration of the entire filter, corrected for the dilution used and original mass of sediment collected. The sum of cell counts on a control slide (just PBS) was subtracted from each slide’s counts to correct for contamination.

### 2.7 Sulfate

Sulfate was measured via ion chromatography (IC) equipped with a 4 mm × 250 mm IonPac AS18 hydroxide-selective anion-exchange column (Dionex ICS-2100). Replicate standards averaged ±0.01% at 20 mM. KOH was used as the eluent with each sample’s retention time set at 24 min. Chloride peaks were also measured with this method, and no abnormalities were observed.

### 2.8 Sulfide

Hydrogen sulfide, the sum of H_2_S, HS^−^, and S^2−^, was measured with an adapted Cline assay to react porewater hydrogen sulfide with Fe^3+^ and diamine to create methylene blue (Cline, 1969). The samples incubated at room temperature for at least 20 min in the dark before a NanoDrop 2000C spectrophotometer measured the absorbance at 667 nm. This instrument has a ± 3% absorbance accuracy.

### 2.9 DIC/ΣCO_2_

Dissolved inorganic carbon (DIC), the sum of CO_2_, HCO_3_^−^, and CO_3_^2−^, was measured only on CLB cores 1–3 using a cavity ringdown spectrometer (Picarro G2201i) at the Chesapeake Biological Laboratory, Maryland. Instrument precision was ±1‰. Samples were acidified with 0.1 mL of 10% HCl so DIC was converted to CO_2._ Vials were injected with 5 mL of zero air and shaken for 2 min, assuming 96.9% of CO_2_ was extracted based on the CO_2_ solubility (Weiss, 1974). Headspace CO_2_ was diluted (5 mL headspace: 135 mL zero air) and injected directly into the spectrometer. If out of the instrument’s range (~2,000 ppm CO_2_), samples were further diluted (35 mL original dilution: 105 mL zero air). Due to the high sulfide concentrations in the porewater, we verified that there was no interference with the CO_2_ signal using a copper trap (Malowany et al., 2015). If no DIC was measured for a sample, the average concentration of the sample above and below was used for thermodynamic calculations.

### 2.10 DIC: sulfate ratios

Ratios of DIC to sulfate were calculated from our measured DIC and sulfate for CLB and from a WOR dataset of two 2013 cores from the same location as our cores, Station H (Steen, 2016). The ratio uses the change in DIC compared to the overlying water column for WOR or 12 cmbsf concentrations for CLB versus the absolute value of the change in sulfate compared to the overlying water column for WOR or 12 cmbsf for CLB. Only depths deeper than the bioirrigation zone where sulfate is held near constant (>12 cmbsf for CLB, >0 cmbsf for WOR) but also in the sulfate reducing zone (<40 cmbsf for CLB, <47 cmbsf for WOR) were used for determination of the slope of the DIC to sulfate stoichiometric ratio. A 2:1 ratio of ∆DIC to ∆SO_4_^2−^ represents OSR and a 1:1 ratio represents sulfate reduction via AOM.

### 2.11 Gibbs energy calculations

The Gibbs energy of reaction, ∆*G_r,_* was calculated for hydrogenotrophic methanogenesis, Equation 6,

(6) $${{HCO}_{3}}^{-}+4H_{2}+H^{+}\to {CH}_{4}+3H_{2}O$$

and sulfate reduction, Equation 7,

(7) $${{\mathrm{SO}}_{4}}^{2-}+4H_{2}+H^{+}\to {HS}^{-}+4H_{2}O$$

using Equation 8:

(8) $$\Delta G_{r}=\Delta G_{r}^{{}^{\circ}}+\mathrm{RTln}Q_{r}$$

where ∆*G*^°^*_r_* refers to the standard-state Gibbs energy of reaction and *Q_r_* is the reaction quotient. It is calculated using Equation 9:

(9) $$Q_{r}=\underset{i}{\prod }a_{i}^{v_{i}}$$

where *a_i_* refers to the activity and *v_i_* is the stoichiometric coefficient of the *i*th species.

The concentrations of CH_4_, H_2_, and DIC were measured in this study as noted above. Since DIC was not measured for WOR, values were used from Kelley et al. (1990). The ratio of bicarbonate to carbon dioxide was calculated to be 0.94 from calculated *in situ* equilibrium constants (Roy et al., 1993). Activity coefficients of CH_4_ and H_2_ were assumed to be 1 and was calculated for bicarbonate as 0.661 in CLB and 0.660 in WOR based on assumed ionic strength of 0.7 M using the CHNOSZ package for R (Dick, 2019), which implements the revised HKF equation of state for Gibbs energies (Shock et al., 1992; Tanger and Helgeson, 1988) and the extended Debye-Hückel equation for activity coefficients (Helgeson, 1969). Values of ∆*G*^°^*_r_* for hydrogenotrophic methanogenesis at CLB (*T* = 24°C, *p* = 1 atm) and WOR (*T* = 28.5°C, *p* = 1 atm) were calculated to be −229.59 kJ/mol and −238.98 kJ/mol, respectively, using the CHNOSZ package.

### 2.12 DNA extraction and sequencing

DNA was extracted using QIAGEN’s RNeasy Powersoil Total RNA Kit with the RNeasy Powersoil DNA Elution Kit, since this kit has been shown to remove co-extracted humic acids, even if the goal is not RNA extraction (Lloyd et al., 2010). All steps in the protocols were followed, using 2 g of sediment, with the following modifications: four freeze thaw steps at 65°C were conducted after step 2. During step 9, samples were incubated at room temperature using a hybridization oven kept at slow rotation, followed by an overnight incubation at 8°C. The V4 region of the 16S rRNA gene was amplified using the Earth Microbiome Project (EMB) 16S Illumina amplicon protocol and the Caporaso 515F and 806R primers. Samples were prepared with the Illumina DNA prep kit and sequenced with an Illumina MiSeq at the Genomics Core at the University of Tennessee.

### 2.13 Data analysis

16S rRNA gene assays were analyzed in R (R Core Team, 2021; RStudio Team, 2020) with version 1.16 of the Divisive Amplicon Denoising Algorithm (DADA2) pipeline (Callahan et al., 2016). Poor read quality samples were removed along with ASVs with less than 5 reads. Contaminants were removed from analysis if eukaryotic or previously identified as contaminants (Sheik et al., 2018). Taxonomy was assigned with version 138.1 of SILVA reference sequences (Quast et al., 2013; Yilmaz et al., 2014). No species level identification was assigned. The resulting loss per each step of analysis is shown in Supplementary Table S2.

The phyloseq package was used for beta diversity and handling of the large data frame (McMurdie and Holmes, 2013). Plots were created primarily with the ggplot2 package (Wickham, 2016). Raw sequences have been reposited in the ENA bank under project ID PRJEB74703. All code is on GitHub at https://github.com/gagecoon/clb23, with the various helper packages used throughout all data analysis listed as imported libraries in the code.

## 3 Results

### 3.1 Geochemistry and Gibbs energy in White Oak River estuary

**Figure 1:**
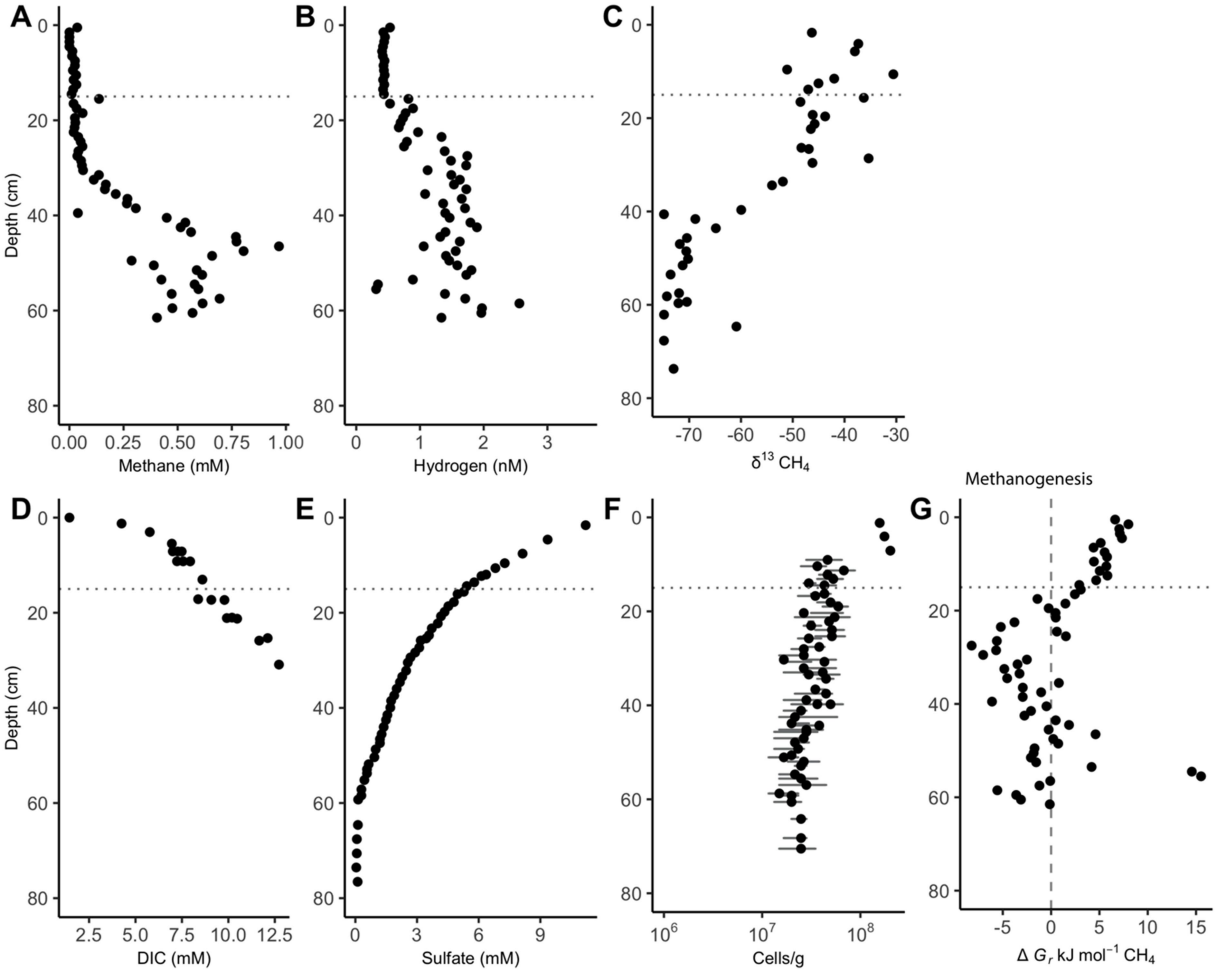
White Oak River estuary’s downcore porewater concentrations of **(A)** methane and **(B)** hydrogen, **(C)** δ13CH4, **(D)** dissolved inorganic carbon (DIC), **(E)** sulfate, **(F)** cells, and **(G)** Gibbs energy values for hydrogenotrophic methanogenesis, ∆Gr, (Reaction 6). The dashed vertical line at 0 kJ/mol delineates equilibrium for Reaction 6, where neither the forward nor reverse reaction is exergonic. The horizontal dotted line at 15 cm is the depth where hydrogen begins to accumulate. Positive values of ∆Gr indicate that AOM via reverse methanogenesis is exergonic while negative values show that hydrogenotrophic methanogenesis is exergonic. Data for subplots C, E, and F is from Kevorkian et al. (2021), and data for subplot D is from Kelley et al. (1990). Cell abundance error bars represent the standard deviation of 30 random counts.

Methane remains low (<0.1 mM) in the upper 30 cm of sediment until it increases and remains between 0.25 and 1 mM between 36 and 62 cm (Figure 1A). The upward curvature of the methane concentrations signifies methane removal through AOM as methane diffuses upward through the core, as has been consistently observed previously (Kevorkian et al., 2021; Lloyd et al., 2011; Martens et al., 1998). Hydrogen remains low (range = 0.41–0.52 nM H_2_) and constant (variance = 0.00081 nM H_2_) for the 15 measurements in the upper 15 cm. Below the upper 15 cm, hydrogen concentrations increase to a range of 0.31–2.56 nM between 20 and 62 cm, showing increased variability (variance = 0.203 nM) (Figure 1B). Hydrogen increases 15 cm above the point where methane begins to accumulate. AOM via Reaction 6 is exergonic in the upper 20 cm while methanogenesis is exergonic from 20 to 40 cm (Figure 1C). Below this point, values are not consistently exergonic in either direction.

### 3.2 Geochemistry and Gibbs energy changes in Cape Lookout Bight

**Figure 2:**
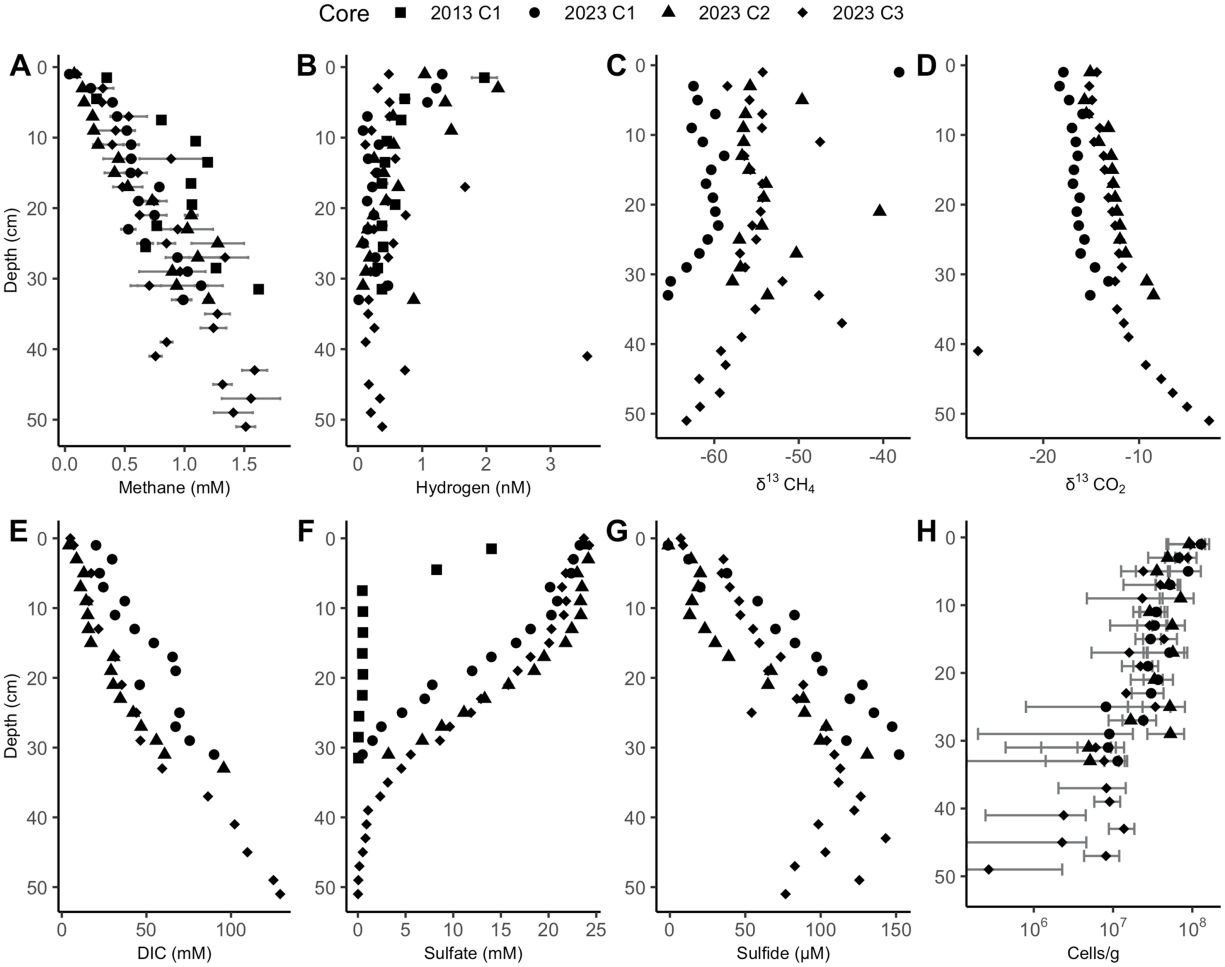
Cape Lookout Bight sediment downcore concentrations of **(A)** methane, **(B)** hydrogen, **(C)** δ13CH4, **(D)** δ13CO2, **(E)** dissolved inorganic carbon (DIC), **(F)** sulfate, **(G)** sulfide, and **(H)** cells. Methane and hydrogen error bars represent triplicate measurements of the same sample. Cell abundance error bars represent the standard deviation of 30 random counts. Only a subset of these measurements was performed for the 2013 core.

Sulfate depletion depths in the Cape Lookout Bight sediments range from 30 to 40 cm, as has been observed previously (Coon et al., 2023; Hoehler et al., 1994), except for the 2013 core which has shallower sulfate depletion. In all CLB cores, methane increases linearly with depth and is not prevented from accumulating in the sulfate-rich upper ~30 cm, suggesting no net removal or production, in agreement with previous geochemical studies at CLB (Hoehler et al., 1994; Martens et al., 1998). Methane concentrations increase to more than 1 mM in 2023 core 3, reaching full saturation (~1.5 mM, Figure 2A). Hydrogen is highly variable, peaking around 2 nM in the upper few cm, and decreasing to less than 1 nM below 10 cm (Figure 2B). δ^13^CH_4_ values range from −60‰ to −66‰ (Figure 2C), decreasing with depth below 24 and 30 cm in 2023 cores 1 and 3, respectively, suggesting deep AOM that does not occur in the upper sections where sulfate and methane are both abundant. The highly negative isotope ratios show upward-diffusing methane is likely methanogenic in origin. The DIC concentrations (Figures 2D,E), which increase linearly with depth to more than 100 mM, are much higher than values measured previously at WOR (Kelley et al., 1990), as expected for having more labile organic matter at CLB. Sulfate decreases with depth from 24 mM to near 0 mM by 10 cm in 2013’s core and at 30–40 cm for 2023’s cores (Figure 2F). Sulfide increases with depth until about 30 cm (Figure 2G). Cell abundance ranges from 10^6^ to 10^8^ for 2023’s cores and slightly decreases with depth in all cores (Figure 2H). Porosity is mostly between 0.7 and 0.85 and decreases slightly with depth in all cores (Supplementary Figure S2).

**Figure 3:**
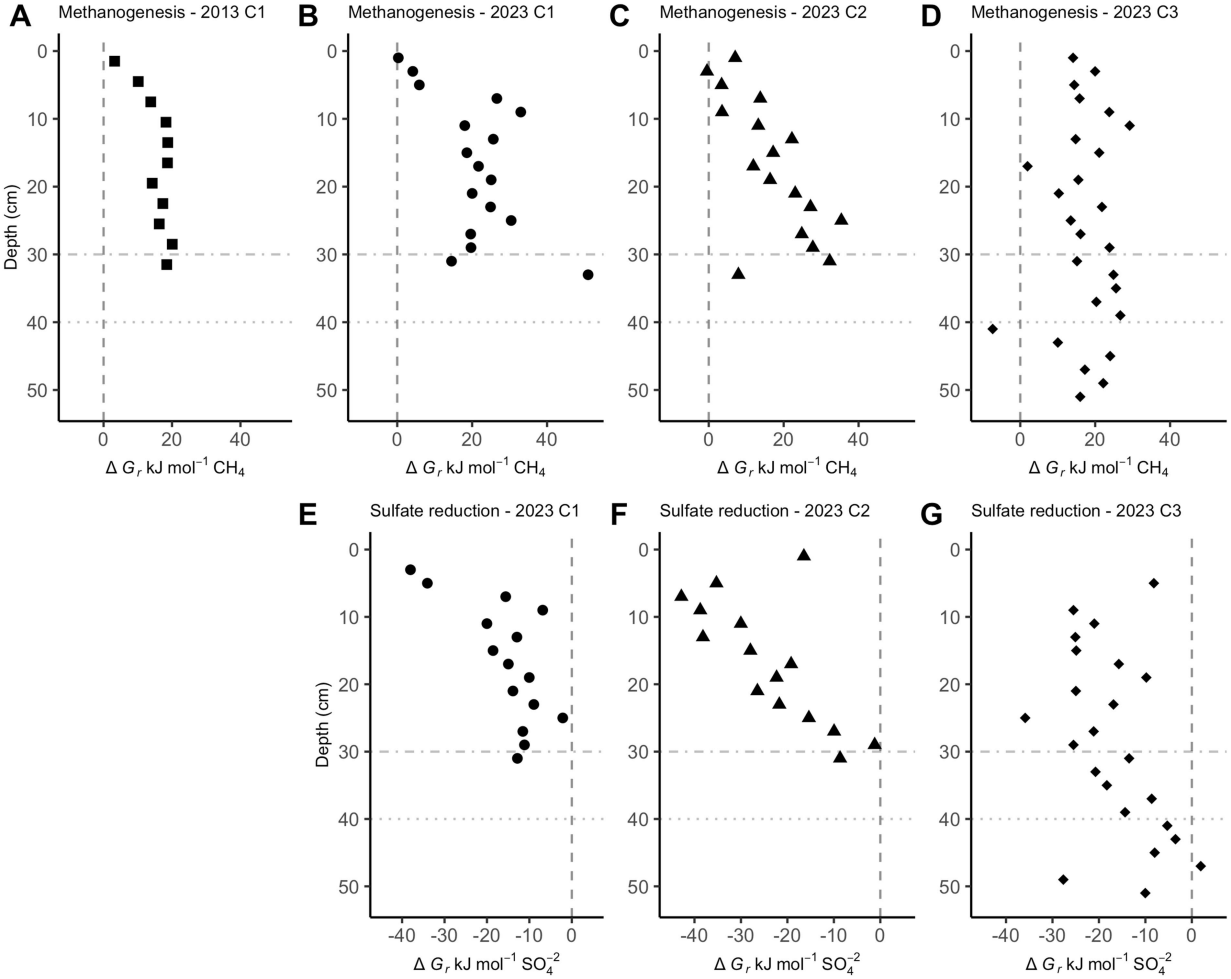
Gibbs energies of reaction, ∆*Gr*, for hydrogenotrophic methanogenesis (**A–D**, Reaction 6) and sulfate reduction (**E–G**, Reaction 7) in Cape Lookout Bight sediments. Values left of the dashed vertical lines at 0 kJ/mol show where these reactions begin to be exergonic. Horizontal dot-dash and dotted lines are where sulfate is depleted in core 1 and core 3, respectively.

Below a few centimeters sediment depth in CLB cores, Gibbs energies are only exergonic for reverse hydrogenotrophic methanogenesis, Reaction 6 (Figure 3A–D). Sulfate reduction, Reaction 7, is exergonic at most depths, ranging mostly from −40 to −10 kJ/mol (Figure 3E-G).

### 3.3 DIC: sulfate ratios

**Figure 4:**
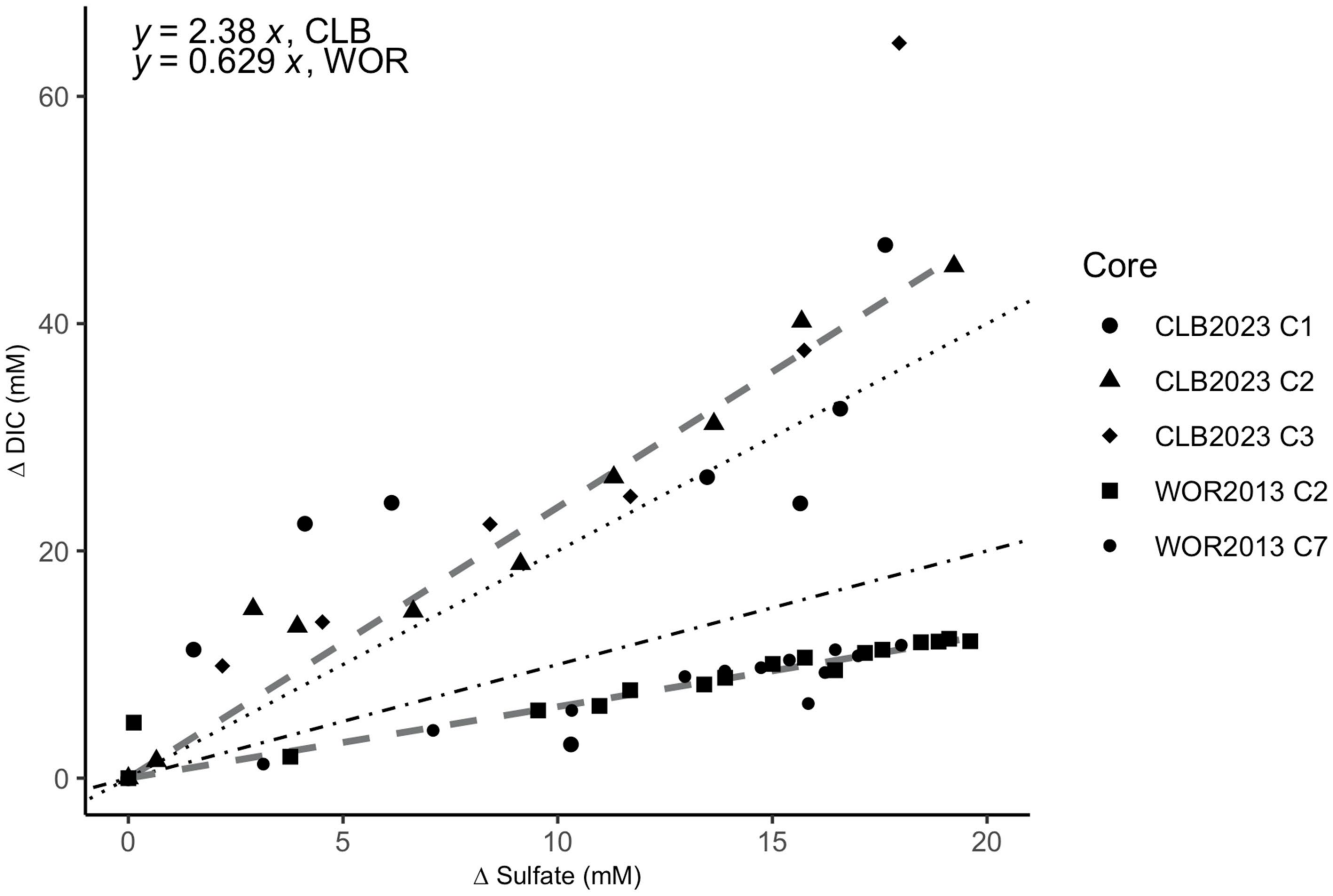
Stoichiometric ratios of the change (∆) in dissolved inorganic carbon (DIC) vs. sulfate in pore fluids from the sulfate reducing zone in Cape Lookout Bight (12–40 cm; circle, triangle, and diamond shapes) and White Oak River estuary (0–47 cm; square and small circle shapes). The change (∆) in DIC vs. sulfate has a ratio of 2.38 for CLB and 0.629 for WOR calculated from linear fits. The dotted line represents the 2:1 ratio of organoclastic sulfate reduction (OSR) while the dot-dashed line represents the 1:1 ratio of AOM via sulfate reduction.

The ratio of ∆DIC to ∆SO_4_^2−^ shows slopes of 2.38 for CLB and 0.629 for WOR (Figure 4). This suggests that organic matter drives sulfate reduction in CLB and AOM drives sulfate reduction in WOR.

### 3.4 Microbial diversity and composition in Cape Lookout Bight

Of the 10,232 observed amplicon sequence variants (ASVs), 84.5% are Bacteria, while 15.5% are Archaea. Non-metric multidimensional scaling (NMDS), principal coordinates analysis (PCoA), and canonical correspondence analysis (CCA) ordination of Bray–Curtis dissimilarity distances show depth is a driving factor in the diversity of microbial life in CLB sediments (Supplementary Figures S3, S4), in accordance with what has been found previously (Coon et al., 2023).

**Figure 5:**
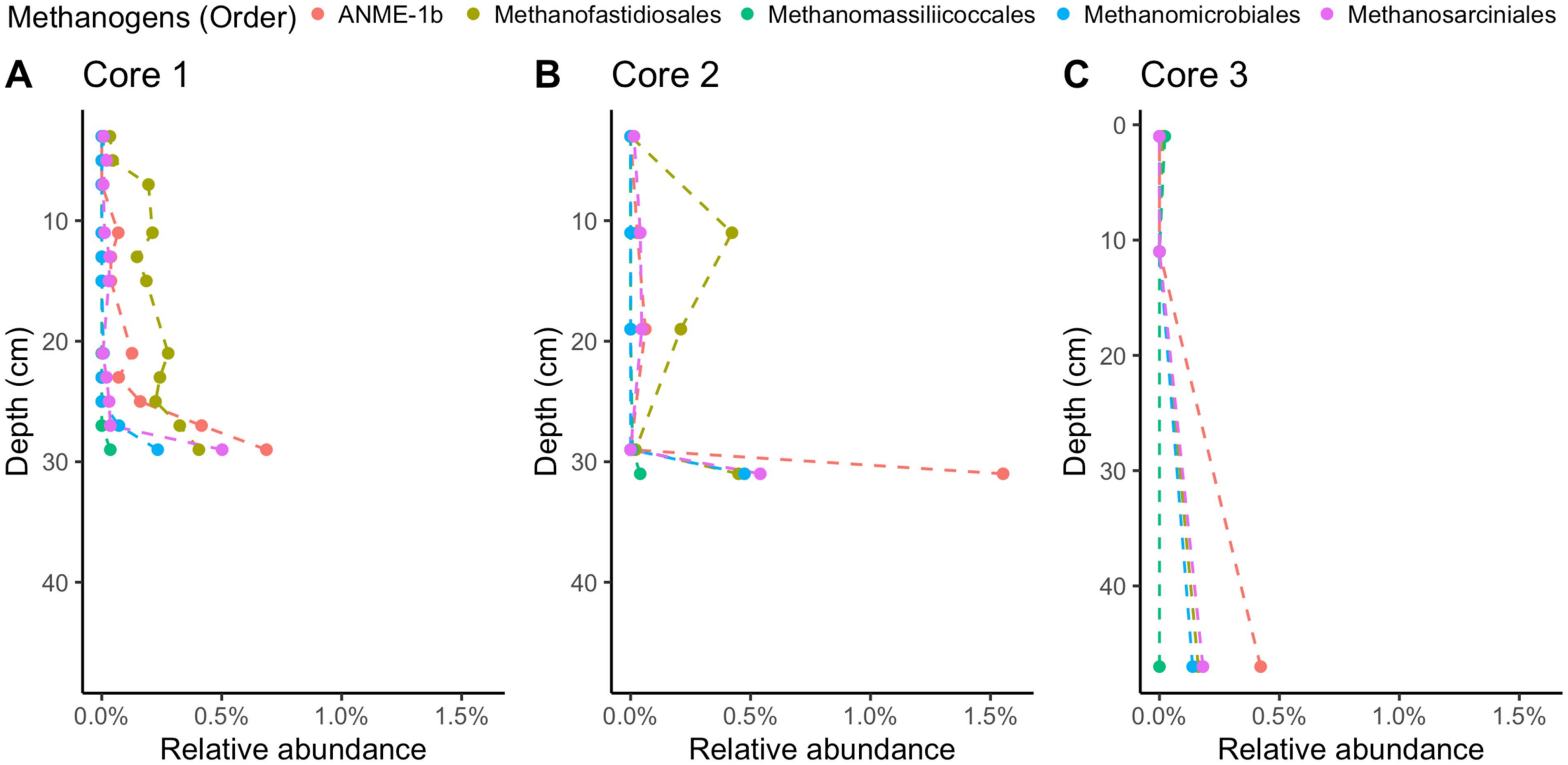
16S rRNA gene amplicon relative abundances for likely methane-cycling archaea in Cape Lookout Bight 2023 cores **(A)** 1, **(B)** 2, and **(C)** 3. Dashed lines are intended to guide the eye.

**Figure 6:**
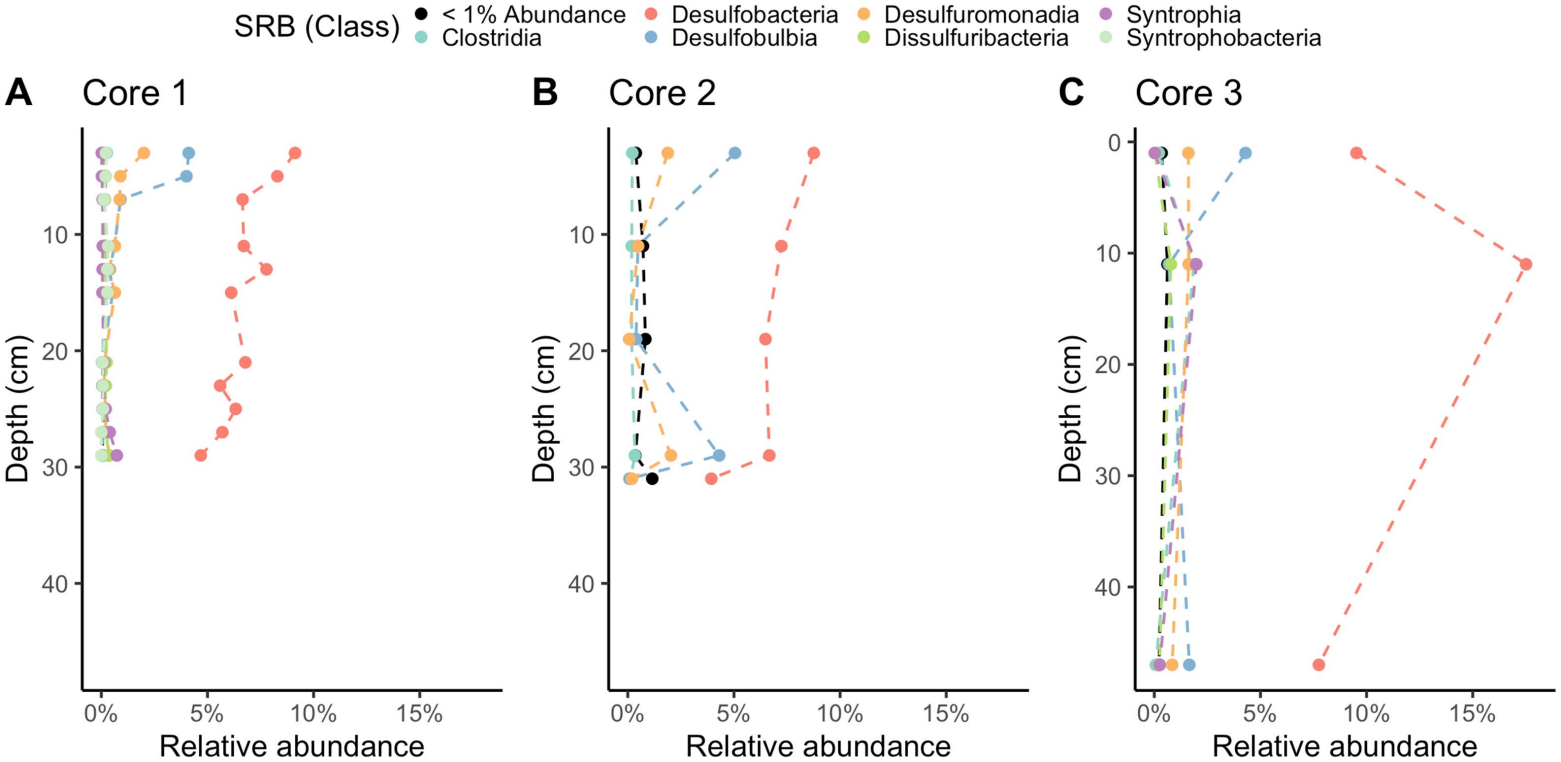
16S rRNA gene amplicon relative abundances for likely sulfate-reducing bacteria (SRB) in Cape Lookout Bight 2023 cores **(A)** 1, **(B)** 2, and **(C)** 3. Dashed lines are intended to guide the eye.

Methane-cycling archaea like *ANME-1b*, *Methanofastidiosales*, *Methanomassiliicoccales*, *Methanomicrobiales*, and *Methanosarciniales* are present throughout the cores (Figure 5), in agreement with previous results (Coon et al., 2023). There is a higher relative abundance of *ANME-1b* than other methane-cycling archaea at the lowest depths (29–31 cm) where sulfate is low (~1.5–3.2 mM) (Figure 5). This sharp increase in ANME-1 near the depth of sulfate depletion has been observed previously (Coon et al., 2023) and matches the pattern observed in the White Oak River estuary (Lloyd et al., 2011; Kevorkian et al., 2021). In sulfate-rich sediments, methanogens capable of using methylated compounds are abundant, *Methanofastidiosales* and *Methanomassiliicoccales* (Figure 5). The majority of likely SRB are *Desulfobacteria*, ranging from 4 to 18% of total abundance (Figure 6). Overall, SRB slightly decrease in abundance with depth as previously observed (Coon et al., 2023).

## 4 Discussion

### 4.1 Molecular hydrogen from fermentation controls the net direction of methane cycling through consortia between SRB and fermentative microbes

Downcore profiles of hydrogen concentration differ greatly between the relatively organic-poor White Oak River estuary and the relatively organic-rich Cape Lookout Bight. In WOR sediments, hydrogen is tightly controlled in the 15 measurements made in the upper 15 cm (variance = 0.00081 nM H_2_). Since this is the depth range where sulfate reduction rates are highest (as shown by the greatest rate of sulfate decrease with depth), a feature that is consistent across years and seasons (Kelley et al., 1990; Lloyd et al., 2011; Martens et al., 1998), these concentrations are likely the result of consistent syntrophy between sulfate reducing microbes and fermentative microbes. Given the consistency of the hydrogen control, it is likely that this is an obligate syntrophy driven by maintaining thermodynamic yields for fermenters degrading recalcitrant lignin-derived compounds dominating the WOR site (Martens et al., 1998). As sulfate is depleted with depth, hydrogen concentrations increase and become highly variable over the 41 measurements made below 20 cm (range = 0.31–2.56 nM H_2_, variance = 0.203 nM H_2_), likely because sulfate becomes diffusion-limited, so sulfate reducers are no longer a reliable syntrophic partner with fermenters. Notably, this occurs well above the depth where net methane production occurs. It coincides with a small gradual increase in methane with depth indicative of net removal of methane diffusing up from below. This was also observed in incubations of WOR sediment where hydrogen increased before methanogenesis started (Kevorkian et al., 2022). It is only when hydrogen concentrations stabilize at a still variable but slightly higher value (1.49 nM H_2_ ± 0.44 nM H_2_) below 30 cm that net methane production occurs. This is the mechanism commonly assumed to occur in anoxic marine sediments; sulfate reducers keep hydrogen concentrations low through syntrophy with fermenters when sulfate is plentiful but lack syntrophy when sulfate is depleted, allowing for higher and more variable hydrogen concentrations and therefore methanogenesis. The only surprising part is that sulfate’s control of hydrogen is released well before net methanogenesis occurs and well within the AOM zone. This agrees with observations of long-term incubations from the same site where hydrogen increases before methanogenesis occurs (Kevorkian et al., 2022).

CLB has a very different hydrogen profile; hydrogen concentrations are never well-controlled (variance = 0.2285 nM H_2_ for 0–30 cm), suggesting a lack of a well-developed obligate syntrophy between sulfate reducers and fermenters. This lack of widespread syntrophy is likely due to the plentiful and highly reactive organic matter in CLB, as has been found previously (Martens et al., 1998). Here, we show that hydrogen concentrations are significantly higher in the upper 10 cm than below it (0.876 nM H_2_ vs. 0.410 nM H_2_, t-value = −2.8945, *p*-value = 0.0077, df = 25.604), which implies that the most labile organic matter—toward the surface—supports the highest hydrogen concentrations. This hydrogen profile with higher abundances at the surface has also been observed in the highly reactive organic matter of the Namibian coast (Lin et al., 2012). Sulfide concentrations validate the observed sulfate profile via the opposite trends; these are further used for the Gibbs energy calculations. In CLB, evidence for net AOM only appears deeper than 35 cm (as seen in the δ^13^CH_4_ and δ^13^CO_2_ profiles), suggesting that labile organic matter needs to be depleted so hydrogen concentrations can decrease and AOM can occur.

Evidence for the difference in reliance on consortia between CLB and WOR appears in the ∆DIC:∆SO_4_^2−^ values, where WOR stoichiometric coefficients reflect sulfate-dependent AOM, and CLB shows organic matter powers sulfate reduction via OSR (Figure 4). The ratio values slightly higher than 2:1 in CLB may reflect excess DIC production via anaerobic heterotrophy and values slightly lower than 1:1 for WOR may reflect net autochthonous carbonate precipitation in WOR. The oxidation state of the organics being non-neutral could also explain the non-integer ratios (LaRowe and Van Cappellen, 2011). Other studies have shown low organic matter lability, like in WOR, promotes sulfate dependent AOM (Pohlman et al., 2013). The OSR in CLB may decrease the favorability of consortia formation between SRB and fermenters since organic matter is more labile. Additionally, with the lack of hydrogen usage, there is the potential for higher and more variable hydrogen concentrations in the organic-rich marine sediment. This would lead to the perceived “messiness” observed in downcore hydrogen measurements that is especially prevalent in the shallowest and most organic-rich depths.

Hydrogen profiles in WOR are consistent with the net AOM observed. WOR has clear removal of methane from AOM [shown via the concave up methane profile, the 1:1 ∆DIC:∆SO_4_^2−^ slope, previous δ^13^C ratios and modeling (Kevorkian et al., 2021, 2022; Lloyd et al., 2011; Martens et al., 1998)]. CLB hydrogen profiles are consistent with the lack of net AOM observed in the upper sulfate-rich sediment (shown via the linear methane profile, the 2:1 ∆DIC:∆SO_4_^2−^ slope, the δ^13^C ratios, and previous studies) (Hoehler et al., 1994; Martens et al., 1998), and AOM below ~35 cm (shown via the δ^13^C ratios and Δ*G_r_*). We conclude that hydrogen does control the net direction of methane-cycling and this hydrogen is controlled by organic matter reactivity. Further, it seems that hydrogen concentrations are controlled by the presence or lack of syntrophy between SRB and fermenters. This has important implications for our understanding of methane cycling from biotic sources in anoxic marine sediment—highly reactive organic matter sites may not have significant methane removal through AOM (Lapham et al., in review; Hoehler et al., 1994, 1998).

### 4.2 Differences in geochemical processes between these two sites are not due to different microbial communities

The difference in metabolic processes between these two sites is likely not due to the presence of different microbial communities. The microbial communities at each site have many of the same key taxa despite their different geochemistry. Compared to previously published 16S rRNA gene sequence abundances from WOR (Kevorkian et al., 2021), the two sites have similar communities of sulfate reducing bacteria, dominated by *Desulfobacteria*. In comparing the methane-cycling archaea, both have a rapid increase of *ANME-1* in the deepest sampled depths where sulfate is at its lowest. There are some *Methanofastidiosales* in WOR, but they are not as consistent in abundance as at CLB. The similarity in microbial composition patterns in the WOR and CLB suggest that the quality of the organic matter has a larger effect on the respiratory processes than the microbial taxa that are present. However, additional methanol sources may promote methylotrophic methanogenesis in CLB, as plant decay and phytoplankton both supply methanol to marine sediments (Bates et al., 2021; Mincer and Aicher, 2016), which may account for the methylotrophic methanogens in our 16S rRNA libraries in CLB.

### 4.3 Observed ∆*G_r_* values underestimate favorability assuming a ∆*G_min_*

The Gibbs energy calculations shown in Figure 1 reveal that sulfate driven AOM and methanogenesis often yield less energy than the ∆*G_min_* of −10 kJ/mol in WOR. However, the geochemical data gathered and analyzed in this study strongly suggests that these processes are occurring where there is not enough energy to satisfy the presumed Δ*G_min_*. It has been shown that metabolic reactions via syntrophic associations have been shown to occur close to thermodynamic equilibrium (∆G ≈ 0 kJ/mol) which could allow for these reactions to be exergonic without reaching the −20 to −10 kJ/mol threshold (Jackson and McInerney, 2002). One explanation is that a ∆*G_min_* does not exist in these sediments, rather life is limited by the rate at which this energy is delivered, or power (LaRowe et al., 2012; LaRowe and Amend, 2015a, 2015b, 2020). It is intuitive that the rate of energy delivery is more important for life rather than the size of the energetic package, since even 10 kJ/mol would be insufficient to support life if only one mole of a reactant were processed over the lifetime of an organism, as an extreme example.

As a though experiment, suppose that a microbially catalyzed reaction could yield 10 kJ/mol (i.e., Δ*G_r_* = −10 kJ/mol) and the Δ*G*_min_ term is more negative than this. According to Equation 10, quantifying the Gibbs energy of the proton motive force (*pmf*),

(10) $$\Delta G_{pmf}=-\mathrm{nF\Delta \Psi}+2.303\mathrm{RT\Delta pH}$$

0.63 moles of protons could be translocated across energy-transducing membranes (solving for *n* for typical values of the other parameters in this equation, Δ*Ψ* = 120 mV, Δ*pH* = −0.5 at 25°C). The assumption that a Δ*G*_min_ must be overcome means that despite this large flux of protons, not a single molecule of ATP could be made from the microbes catalyzing this hypothetical reaction, despite the 3.79 × 10^23^ protons (*N_A_* * 0.63 mol H^+^) passing through their membranes. If this *ΔG_min_* were not assumed to exist, 10 kJ per reaction turnover could, if maximally utilized, yield about 0.17 *moles* of ATP under the specified conditions, which is a clearly sufficient amount of ATP to sustain life.

The ∆*G_min_* was first stated as an unreferenced assumption that has been perpetuated as conventional wisdom (Hoehler, 2004; Hoehler et al., 2001; Schink, 1990, 1997, 2002; Schink and Stams, 2006; Schink and Thauer, 1988). In the original paper exploring the energetics of anaerobic sludge degradation, (Schink and Thauer, 1988), observed that (a) butyrate fermentation yields “20–25 kJ per mol partial reaction,” (b) 75 kJ are required to synthesize 1 mol of ATP and (c) three protons must pass through an energy-transducing membrane to make *one molecule* of ATP. They combine this information, to state, “[t]hus, the equivalent of 1 transported proton is the smallest amount of energy which can be converted into biologically useful energy, meaning: into ATP synthesis.” Not only are the values of ∆*G_ATP_* production at least 25% higher than what is accepted today [75 vs. 60 kJ (mol ATP)^−1^, though they are variable given the particular temperature, pressure, and compositional conditions—(Larowe and Helgeson, 2007)], the authors have assumed that the energy from fermentation is split evenly between the three groups of organisms involved in butyrate fermentation, despite the fact that the energetics of the intermediate reactions being catalyzed are not equal. The work of many others have addressed an alternative to ∆*G_min_* by using a minimum maintenance energy over time, i.e., power, to describe minimum energy thresholds for microbial life (Hoehler and Jørgensen, 2013; Tijhuis et al., 1993). Power has been used instead of just ∆*G_r_* to better constrain the lower limits of energy usage in natural settings (Bradley et al., 2020, 2022; LaRowe et al., 2020a; Zhao et al., 2021). These works show power is a more apt metric for determining energy limits for microbial life. Our data from WOR suggest that a ∆*G_min_* does not exist because the direction of methane production or consumption changes with the sign of ∆*G*, rather than the crossing of a −10 kJ/mol threshold, which is also supported by theory (LaRowe et al., 2012) and the lack of a consensus ∆*G_min_* in the literature.

CLB sediment has been shown to lack AOM in the presence of sulfate through radiotracers, geochemical profiles, and stable carbon isotope ratios (Hoehler et al., 1994; Martens et al., 1998, and this paper), yet our Gibbs energy changes predict AOM occurring even in the shallow sediment even though it clearly does not occur there (Figure 3). We hypothesize that the large amounts of labile organic matter at CLB mean that the values we measure do not represent the instantaneous values experienced by methane-cycling archaea in close proximity to hydrogen-producing fermenters over small spatial scales.

## 5 Conclusion

**Figure 7:**
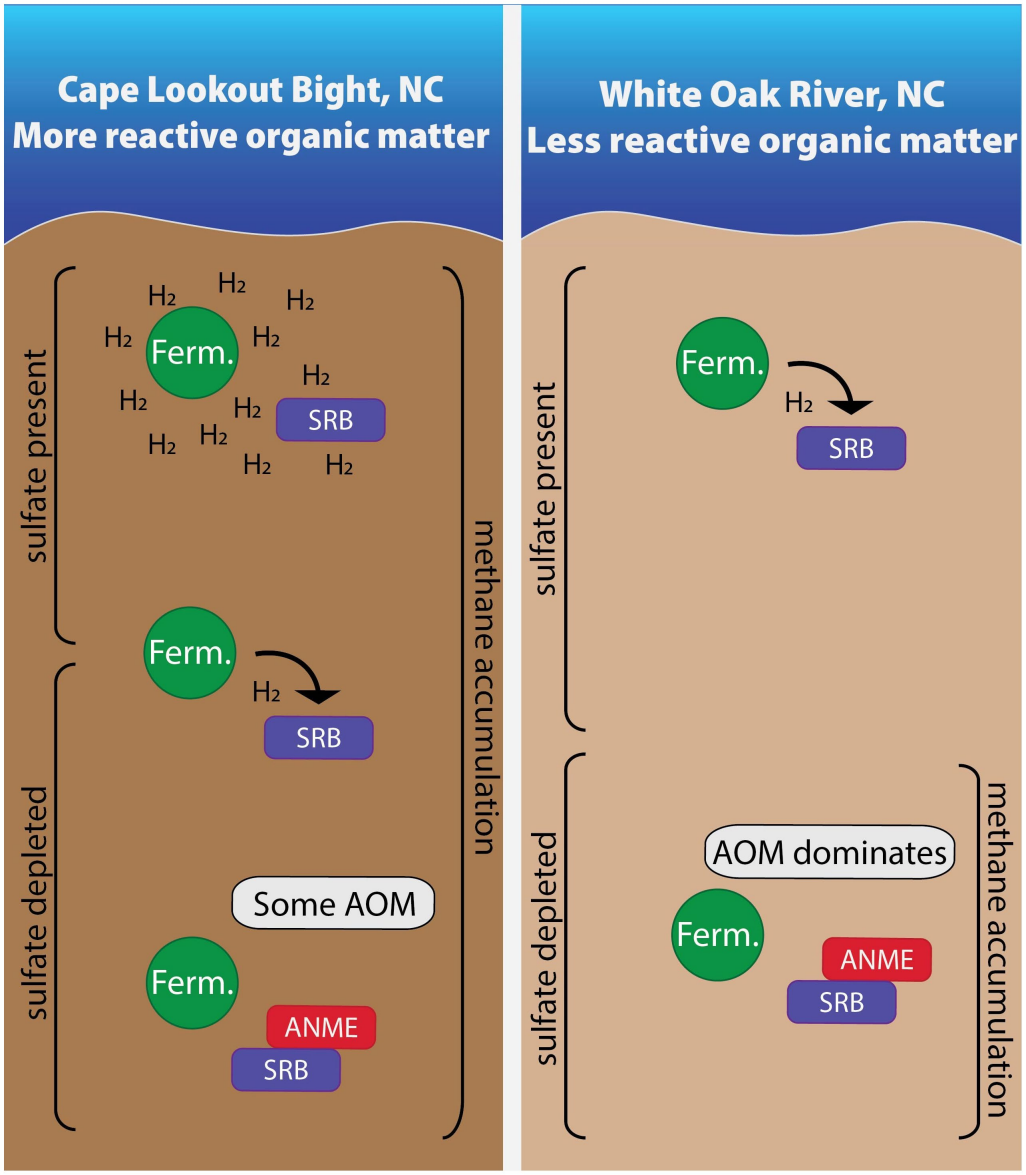
In sites with more reactive organic matter, hydrogen accumulates due to high fermentation rates in shallow areas. As the most labile organic matter is depleted with depth, fermentative microbes and sulfate reducing bacteria energetically rely on one another. Once sulfate is depleted, some AOM occurs in these sites with more reactive organic matter while AOM dominates in the site with less reactive organic matter. Areas are not to scale.

We measured hydrogen concentrations in two sites (WOR and CLB) with different organic matter reactivity and found these values to be useful for understanding the methane cycle in anoxic marine sediment (summarized in Figure 7). Hydrogen concentrations are tightly controlled by sulfate reducers in the presence of poorly reactive organic matter in WOR, allowing AOM in sulfate-rich sediments while hydrogen concentrations are higher and more variable with the highly reactive organic matter of CLB, preventing AOM in sulfate- and methane-rich sediments. However, the concentrations of species in the reactions describing hydrogenotrophic methanogenesis did not always yield values of ∆*G_r_* that exceed what is thought to be a minimum catabolic energy yield that acts as a thermodynamic limit on life, ∆*G_min_*. We have concluded that, in the face of concentration profiles and stable carbon isotopes, that the ∆*G_min_* is not a prerequisite for a lower energetic limit of life, other than the obvious fact that ∆*G_min_* mut be less than 0. As has been discussed elsewhere, perhaps a minimum power limit for life is a more apt metric for determining the energy limits for life. In the context of our samples shown here, ∆*G_r_* is useful for predicting reaction favorability in controlled sites like WOR, assuming there is no ∆*G_min_*. Samples from CLB have higher hydrogen concentrations than WOR; we hypothesize this is due to consortia disruption from the presence of highly reactive organic matter. Due to the difference in organic matter reactivity, CLB and WOR have vastly different hydrogen concentrations and variability with comparable microbial communities. This means that in areas of highly reactive organic matter, net removal of methane through AOM does not occur because the high and variable hydrogen concentrations prevent reverse hydrogenotrophic methanogenesis.

## Data Availability

The datasets presented in this study can be found in online repositories. The names of the repository/repositories and accession number(s) can be found in the article/Supplementary material.
