# Supplementary material for: Control of hydrogen concentrations by microbial sulfate reduction in two contrasting anoxic coastal sediments
Source: Front Microbiol. 2024 Nov 12;15:1455857. doi: 10.3389/fmicb.2024.1455857 (PMC11594503; doi:10.3389/fmicb.2024.1455857)
Supplement: Supplementary file 1 [file Supplementary_file_1.docx]

Supplementary Material

# Supplementary Figures and Tables

## Supplementary Tables

As discussed in Section 2.2 of the main manuscript, the porosity outliers in Supplementary Table 1 were replaced with the surrounding values downcore. This helped us keep methane concentrations more stable so our *∆G_r_* values were clearer to analyze.

| Core | Depth | Porosity | Status | New porosity |
| --- | --- | --- | --- | --- |
| 1 | 3 | 0.7537 | Outlier | 0.7956 |
| 1 | 9 | 0.7440 | Outlier | 0.7998 |
| 3 | 19 | 0.5885 | Outlier | 0.7833 |
| 2 | 21 | 0.5959 | Outlier | 0.7663 |
| 3 | 29 | 0.3315 | Outlier | 0.7336 |
| 3 | 41 | 0.7853 | Outlier | 0.7545 |
| 4 | 41 | 0.7777 | Outlier | 0.7545 |
| 3 | 49 | 0.7091 | Outlier | 0.7191 |

**Supplementary Table 1.** Porosity outliers removed from dataset. See porosity methods for details.

To check how much loss our sequenced reads experienced throughout the dada2 pipeline, we tracked the steps in Supplementary Table 2, showing the read counts in the cells. These samples were high enough quality (>30,000 reads) to proceed with analysis.

|  | **start** | **filtered** | **denoisedF** | **denoisedR** | **merged** | **remove chimeras** |
| --- | --- | --- | --- | --- | --- | --- |
| **Core1_sec10** | 78171 | 71123 | 64626 | 64301 | 55349 | 54578 |
| **Core1_sec11** | 92443 | 84935 | 78895 | 78668 | 70728 | 70101 |
| **Core1_sec12** | 83904 | 76201 | 70570 | 70238 | 62091 | 61500 |
| **Core1_sec13** | 95298 | 80147 | 74444 | 74436 | 66684 | 65942 |
| **Core1_sec14** | 43090 | 40706 | 36612 | 36202 | 31400 | 31021 |
| **Core1_sec15** | 47448 | 42071 | 38315 | 37494 | 31377 | 31123 |
| **Core1_Sec2** | 165076 | 141018 | 128543 | 128088 | 108434 | 107183 |
| **Core1_sec3** | 69672 | 64885 | 57695 | 57470 | 49498 | 49188 |
| **Core1_sec4** | 134887 | 124582 | 116317 | 116143 | 104913 | 104306 |
| **Core1_sec6** | 140661 | 129025 | 120497 | 120031 | 105271 | 103797 |
| **Core1_sec7** | 117468 | 102404 | 94234 | 94230 | 84116 | 82765 |
| **Core1_sec8** | 104591 | 96198 | 88280 | 87896 | 76208 | 75070 |
| **Core2_Sec15** | 164621 | 148323 | 135997 | 135411 | 113803 | 111752 |
| **Core2_sec16** | 135492 | 123496 | 117121 | 117076 | 107531 | 105747 |
| **Core2_Sec2** | 105745 | 94469 | 85438 | 85181 | 71074 | 70175 |
| **Core2_sec6** | 106825 | 98481 | 91322 | 91322 | 81802 | 81272 |
| **Core3_Sec1** | 134771 | 122506 | 137788 | 137530 | 123705 | 120942 |
| **Core3_Sec6** | 27184 | 16593 | 64626 | 64301 | 55349 | 54578 |

**Supplementary Table 2.** Loss per step of DADA2 analysis.

## Supplementary Figures

To ensure our hydrogen methods worked, we checked the same replicate vials for hydrogen concentration over a couple weeks. We found it took at least 13 punctures over 216 hours before it could escape. All of our measurements for the manuscript were taken with this in mind, so done before this 9 day cutoff we observed in Supplementary Figure S1.

**Supplementary Figure S1.** Hydrogen leak test over an elapsed 400 hours, showing hydrogen is contained for at least 216 hours after flushing.

We visualized porosity downcore (Supplementary Figure S2) to ensure the trends match expectation, slowly decreasing with depth and consistent between all cores. This also allowed us to compare the 2013 core with the 2023 cores and ensure the environments are still similar.

For broad scale comparison of the cores, we plotted beta diversity (Supplementary Figure S3) and total phyla distribution (Supplementary Figure S4). As depth groups correlate closer in the beta diversity plots than cores, we determine the three cores are representative of biological replicates and depth is the driving factor for similar microbial communities. These changes in phyla can be seen in S4, where the largest changes are *Desulfurobacterota* and *Proteobacteria* decreases with depth while *Chloroflexi* increases with depth.

**Supplementary Figure S2.** Cape Lookout Bight’s downcore porosity (plot A).

**Supplementary Figure S3.** Beta diversity visualized via NMDS, PCoA, and CCA of Bray-Curtis dissimilarity distances.

**Supplementary Figure S4.** Cape Lookout Bight’s 16S rRNA gene amplicon relative abundances for phyla with more than 1% relative abundance.
